# Dugald Macniel, M. D.

**Published:** 1885-04

**Authors:** 


### Dugald Macniel, M. D.

Died March 21, 1885, Aged 42 Years.

The medical profession of this city have sustained a very severe loss in the death, at a comparatively early age, of Dr. Dugald Macniel. In paying the tribute of respect to his personal and professional worth, we are prompted not only by a deep appreciation of his attainments, but by a friendship, which, from long and intimate association, had ripened into filial love.

The life history of Dr. Macniel affords an instance of success from steadfast devotion to correct principles, and from earnest effort for the attainment of cherished results. He was born in Scotland in 1843, and at an early age emigrated to Canada. On the passage, his father and only brother died and were buried at sea. On her arrival in a strange land, the widowed mother, deprived of her natural protector, was thrown on her own resources for the support and education of her only son. Thus, early, the subject of this sketch was instructed in habits of industry and economy. With his limited advantages he struggled on, and by close application and great industry he secured a fair academic education, after which he entered upon the study of medicine, entering, in 1869, the medical class at the Buffalo Medical College, from which he graduated in 1871. He at once connected himself with the Buffalo Free Dispensing Association, at first as interne, and subsequently as a member of the attending staff, in every position showing the same industry and attention to duty, and as a reward of earnest labor reaping merited success. In 1883 he was elected Lecturer of Clinical Dermatology in the Medical Department of Niagara University. With a view to prepare himself for the position, he went abroad and passed several months in Vienna under the best teachers in that great medical centre. Returning to Buffalo in the autumn of 1884, he at once resumed the practice of his profession, and with the improved advantages and study of his sojourn abroad, he was prepared to reap the reward of years of patient toil and unremitting industry.

Dr. Macniel was a very successful practitioner. He possessed rare judgment, fine professional attainments, tireless energy and industry, a sympathetic nature which won for him the confidence and affection of his patients. The future was peculiarly promising to him. Possessed of ample pecuniary resources, he was prepared to enter upon a career of prosperity, which was to him the reward of labors begun in his early youth and continued without cessation until success had been attained.

We give the action of the Medical Societies of which Dr. Macniel was a member and of the medical faculty to which he belonged.

The following resolutions were passed by the meeting of the Erie County Medical Society, held March 23, 1885:

"WHEREAS, It has pleased God to remove from among us Dr. Dugald Macniel.

"*Resolved*, That we deeply lament the loss of our worthy professional associate and friend.

"*Resolved*, That we desire to record the high appreciation and esteem we feel for the great worth of his public and private character. His was a quiet, unostentatious, busy life. His high moral character and universal goodness of heart procured for him the respect and esteem of his professional friends, his numerous patients and the general community.

"*Resolved*, That we extend our heartfelt sympathy to his bereaved family.

"*Resolved*, That we attend the funeral in a body.

"*Resolved*, That a copy of these resolutions be transmitted to the family and furnished to the medical and daily press for publication."

At a meeting of the faculty of the medical department of Niagara University, held at the college building, Saturday evening, March 21, 1885, the following memorial was adopted:

"Death has entered our midst and claimed one of our number, Dr. Dugald Macniel, lecturer on clinical dermatology. For the first time since our organization we are called upon to mourn the loss of an associate. Our personal attachment for our professional brother makes sorrow doubly sad. One of the secrets of his success in life was the ease with which he made friends. He was a genial and cheerful companion, a physician in the true sense, kind, considerate, faithful and attentive to the sick and to his trust.

"We, the members of the medical faculty, deeply mourn his loss and tender our sympathy and condolence to the bereaved family. We feel that they in their sorrow as well as we in ours should bow to the Divine will."

At a meeting of the Buffalo Obstetrical Society, held March 24, 1885, the President, Dr. W. W. Potter, in announcing the death of Dr. Macniel, made the following remarks, which were ordered spread upon the minutes and made the expression of the society, in commemoration of the event to which they referred:

"This society is, quite early in its history, called upon to mourn the death of one of its members. Though but recently elected to membership, it is more than probable that Dr. Macniel would have proved a valuable addition to our number, his entire professional career having afforded evidence in justification of this prediction. There are others present who can speak of him from the standpoint of intimate acquaintanceship, extending through a long period of years, while my own is comparatively recent; yet my knowledge of his character is sufficient for me to affirm that he was a man who commanded the respect of all who knew him; a physician who performed his duties conscientiously, faithfully and skillfully; a citizen of more than ordinary worth, whose loss will be felt by the community in many ways; a husband and father who has left loving hearts desolate, and which refuse to be comforted."

"The various medical societies, as well as other bodies of which he was a useful member, will suffer great loss in being deprived of his presence, advice and valuable services in their meetings and deliberations; and especially will the young and growing medical college, in which he was a teacher, sustain the loss of a scholarly associate and well-equipped tutor.

"It is not often that the medical profession in this city has been called upon to mourn the death of so young a member, and this seems to add much to the solemnity of the event which we all so deeply deplore. When the old man dies, after having rounded the farthermost goal of life's wearisome race, though grieved we may be when the final summons comes, yet in it we recognize the fulfillment of that inevitable and irrevocable law, which limits and bounds the duration of human affairs and all things earthly. But when a man, and especially a physician and a co-worker in the cause of humanity and science, is cut off in the fullness of his years and the plenitude of his powers, it is an event of more than ordinary significance, and the shock is truly appalling. A few weeks ago Dr. Macniel seemed in the full vigor of health, in the enjoyment of a useful and happy life, and faithfully attentive to the duties of his chosen profession. Now his lifeless body rests within the walls of yonder charnel-house, while his spirit has passed within the portals of that other and better mansion—'that house not made with hands, eternal in the heavens'—to meet, let us hope, the rewards belonging to a life-work nobly and manfully accomplished.

"In his sad demise we are again painfully admonished that—

* * * * 'Life is but a shadow.
Its date the intermediate breath we draw;
Ten thousand accidents in ambush lie,
To crush the frail and fickle tenement,
Which, like the brittle hour-glass measuring time,
Is often broke ere half its sands are run.'

"Let me add my own personal sense of sorrow which this sad event engenders, and let me mingle my tears with yours in extending to the bereaved widow and bereft children, that sympathy and condolence which is as spontaneous as it is heartfelt."

